# Combined Inhibition of Epigenetic Readers and Transcription Initiation Targets the EWS-ETS Transcriptional Program in Ewing Sarcoma

**DOI:** 10.3390/cancers12020304

**Published:** 2020-01-28

**Authors:** Günther H.S. Richter, Tim Hensel, Oxana Schmidt, Vadim Saratov, Kristina von Heyking, Fiona Becker-Dettling, Carolin Prexler, Hsi-Yu Yen, Katja Steiger, Simone Fulda, Uta Dirksen, Wilko Weichert, Shudong Wang, Stefan Burdach, Beat W. Schäfer

**Affiliations:** 1 Children’s Cancer Research Centre and Department of Pediatrics, Klinikum rechts der Isar, Technische Universität München, 80804 München, Germany; hensel.t@hotmail.de (T.H.); oxana.schmidt@tum.de (O.S.); kristina.heyking@tum.de (K.v.H.); f.beckerdettling@gmail.com (F.B.-D.); carolin.prexler@tum.de (C.P.); stefan.burdach@tum.de (S.B.); 2 German Cancer Research Center (DKFZ), partner site Munich, 81377 Munich, Germany; hsi-yu.yen@tum.de (H.-Y.Y.); wilko.weichert@tum.de (W.W.); 3 Division of Oncology and Hematology, Department of Pediatrics, Charité - Universitätsmedizin Berlin, 13353 Berlin, Germany; 4 Department of Oncology and Children’s Research Center, University Children’s Hospital, 8032 Zurich, Switzerland; vadim.saratov@kispi.uzh.ch (V.S.); beat.schaefer@kispi.uzh.ch (B.W.S.); 5 Institute of Pathology, Technical University of Munich and Comparative Experimental Pathology (CEP), Technical University of Munich, 81675 Munich, Germany; katja.steiger@tum.de; 6 Institute for Experimental Cancer Research in Paediatrics, Goethe-University Frankfurt, 60528 Frankfurt/Main, Germany; simone.fulda@kgu.de; 7 Pediatrics III, West German Cancer Centre, University Hospital of Essen, 45147 Essen, Germany; uta.dirksen@uk-essen.de; 8 German Cancer Research Center (DKFZ), partner site Essen, 45147 Essen, Germany; 9 Centre for Drug Discovery and Development and School of Pharmacy and Medical Sciences, University of South Australia Cancer Research Institute, Adelaide, South Australia 5001, Australia; Shudong.Wang@unisa.edu.au

**Keywords:** Ewing sarcoma, tumor growth, CDK9, BRD4, combination therapy

## Abstract

Background: Previously, we used inhibitors blocking BET bromodomain binding proteins (BRDs) in Ewing sarcoma (EwS) and observed that long term treatment resulted in the development of resistance. Here, we analyze the possible interaction of BRD4 with cyclin-dependent kinase (CDK) 9. Methods: Co-immunoprecipitation experiments (CoIP) to characterize BRD4 interaction and functional consequences of inhibiting transcriptional elongation were assessed using drugs targeting of BRD4 or CDK9, either alone or in combination. Results: CoIP revealed an interaction of BRD4 with EWS-FLI1 and CDK9 in EwS. Treatment of EwS cells with CDKI-73, a specific CDK9 inhibitor (CDK9i), induced a rapid downregulation of EWS-FLI1 expression and block of contact-dependent growth. CDKI-73 induced apoptosis in EwS, as depicted by cleavage of Caspase 7 (CASP7), PARP and increased CASP3 activity, similar to JQ1. Microarray analysis following CDKI-73 treatment uncovered a transcriptional program that was only partially comparable to BRD inhibition. Strikingly, combined treatment of EwS with BRD- and CDK9-inhibitors re-sensitized cells, and was overall more effective than individual drugs not only in vitro but also in a preclinical mouse model in vivo. Conclusion: Treatment with BRD inhibitors in combination with CDK9i offers a new treatment option that significantly blocks the pathognomonic EWS-ETS transcriptional program and malignant phenotype of EwS.

## 1. Introduction

Ewing sarcoma (EwS) is a highly malignant bone and soft tissue neoplasia arising predominantly in the pelvis and long bones in children and young adults with early metastasis to lung and bone [1]. EwS is defined by specific balanced chromosomal *EWSR1/ETS* translocations that give rise to oncogenic chimeric proteins, the most common being EWS-FLI1 as a consequence of the t(11;22)(q24;q12) translocation [2,3,4]. Other contributing somatic mutations involved in disease development have only been observed at low frequency [5,6,7,8,9].

EWS-FLI1 acts both as a transcriptional repressor and activator via de novo chromosomal binding mechanisms of the chimeric protein [10,11] accompanied by a specific pattern of histone H3 lysine 27-acetylation (H3K27ac) that are recognized by the BET (bromodomain and extraterminal (ET)) protein family such as BRD2, BRD3, and BRD4. These BRDs are nuclear proteins that carry two bromodomains and an additional ET domain and are implicated in chromatin interactions [12].

BRD4, a prevalent member of the human BET protein family, binds acetylated histones during mitosis to maintain chromatin structure in the daughter cell [13,14]. Specific inhibitors of BET proteins such as I-BET151 or JQ1 result in the displacement of BRDs from chromatin and inhibition of transcription at key genes such as *BCL2*, *MYC*, and *CDK6* [12], and induce growth arrest and differentiation of cancer cells [15,16]. BET inhibitors could block the growth of a number of different malignancies [17]. By the use of JQ1, we significantly blocked proliferation and in vivo tumor growth of different EwS lines and strikingly observed a strong down-regulation of the pathognomonic EWS-FLI1 protein. Subsequent analysis revealed that JQ1 treatment blocked an EwS specific expression program and enhanced apoptosis of EwS [18].

BRD4 facilitates the accessibility of the transcription machinery to specific chromatin regions, ensuring the re-initiation of transcription following mitosis [19]. During transcription pausing, BRD4 recruits the positive transcription elongation factor b (P-TEFb), composed essentially of the cyclin-dependent kinase 9 (CDK9) activated upon its association with T-type cyclins [20]. Two regions of BRD4 directly bind to P-TEFb. The C-terminal domain (CTD) can interact with Cyclin T1 and CDK9, while BD2 recognizes an acetylated region of Cyclin T1 [21,22,23]. P-TEFb phosphorylates RNA Pol II C-terminal domain and promotes transcription elongation [22,24]. The kinase subunit of P-TEFb, CDK9, does not only phosphorylate RNA Pol II but, in addition, phosphorylates the DRB sensitivity-inducing factor (DSIF) and negative elongation factor (NELF), which then dissociate from RNA Pol II [25], finally assuring productive initiation of RNA synthesis [26]. Furthermore, it was recently demonstrated that CDK9 is also essential for maintaining gene silencing at heterochromatic loci [27]. Based on its central role in transcriptional initiation and elongation, CDK9 quickly came into focus to consider its contribution to tumor development and progression [27,28,29], also in pediatric sarcoma [30,31].

The putative interaction of BRD4 with P-TEFb seems therapeutically interesting, especially for a transcriptionally driven tumor such as EwS [10]. In this study, the binding of BRD4 to P-TEFb was confirmed by Co-IP experiments. Treatment of EwS cells with CDK9i induced a rapid down-regulation of EWS-FLI1 expression and reduced contact-dependent growth, as previously observed for BRD inhibition [18]. The transcriptional program following CDK9 inhibition was only partially related to BRD inhibition. However, combined treatment of EwS with BRD and CDK9 inhibitors in vitro and in a preclinical mouse model in vivo overall was more effective than individual drug application.

## 2. Results

### 2.1. BRD4 Interacts with CDK9, and Its Inhibition Promotes the Development of Resistant Cells

Previously, we demonstrated that EwS are susceptible to treatment with epigenetic inhibitors, such as JQ1, blocking BET bromodomain activity and the associated pathognomonic EWS-ETS transcriptional program [18]. In addition, other BET bromodomain inhibitors such as dBET and iBET conferred similar inhibitory activity in EwS (Becker-Dettling, data not shown) [32]. Further reports suggested that cells treated with JQ1 can acquire resistance following sustained treatment [33].

**Figure 1:**
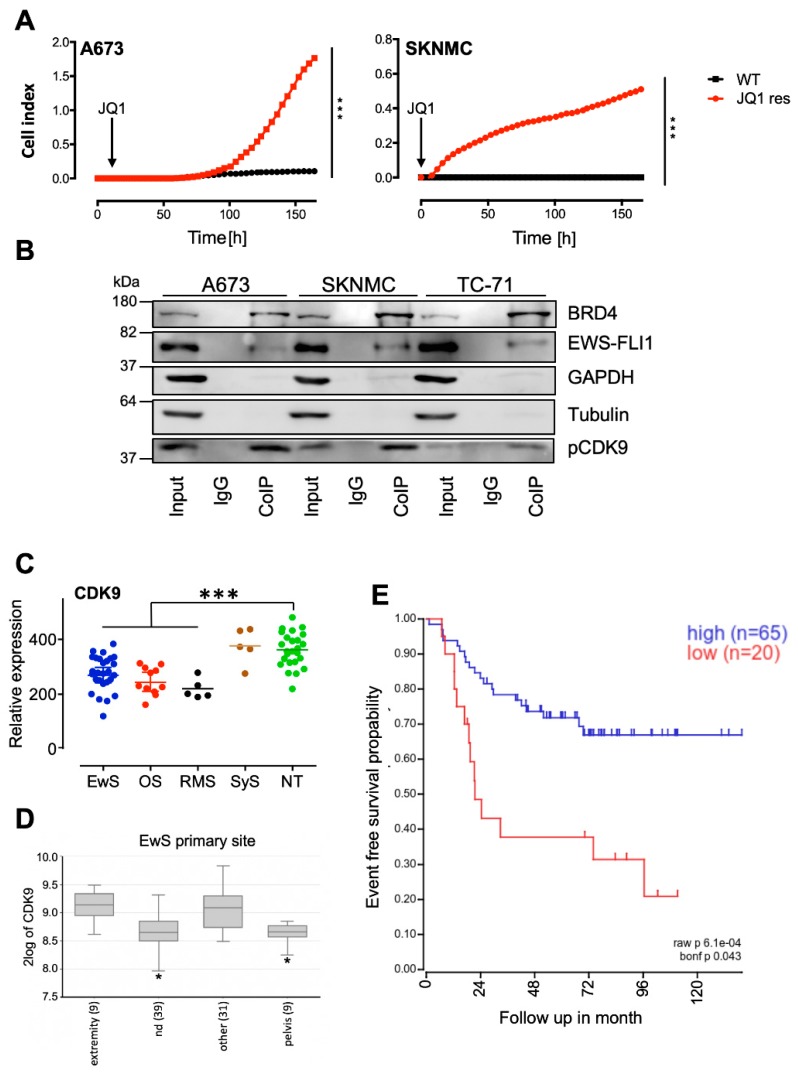
Development of JQ1 resistant Ewing sarcoma (EwS) and interaction with p-CDK9. (**A**) EwS cell lines A673 and SKNMC were treated for 5–6 weeks with 2 µM JQ1 (JQ1 res) and analyzed subsequently by xCELLigence assay. Wild type (WT) A673 and SKNMC cell lines were used as controls. All cells were treated with 2 µM JQ1 during measurement. Cellular impedance was measured every 4 h (relative cell index). Data are mean ± SEM (hexaplicates/group); *t*-test, *** *p*-value < 0.0005. (**B**) Interaction analysis by Co-IP of BRD4, EWS-FLI1, and p-CDK9. Co-IP for A673, SKNMC, and TC-71 cell lines using anti-BRD4 antibodies. After Co-IP, proteins were analyzed by Western blotting for p-CDK9, BRD4, and EWS-FLI1. GAPDH and Tubulin served as a loading control. (**C**) Gene expression profiles of the *CDK9* gene on 27 EwS, 11 osteosarcomas (OS), 5 rhabdomyosarcomas (RMS), 5 synovial sarcomas (SyS), and 25 samples of different normal tissue (NT). Patient RNA were hybridized onto Human Gene ST1.0 arrays (Affymetrix; GSE45544, GSE73166) and compared to a published microarray study of normal tissue (GSE45544). *** *p*-value < 0.0005. (**D**) Differential expression levels of CDK9 in primary EwS at different tumor sites by box plot presentation using the GSE63157 study set and the amc onco-genomics software tool (https://hgserver1.amc.nl/cgi-bin/r2/main.cgi.). The number of samples in each cohort is given in brackets. * *p*-value < 0.05 (**E**) CDK9 expression correlates with event-free survival: Kaplan–Meier estimates for event-free survival probability for CDK9 expression (*n* = 85, *p* = 0.043), GSE63157 study set.

Here, we also confirmed that the treatment of EwS cell lines with normal concentrations of JQ1 for more than 5-6 weeks results in the development of resistant cell lines, as demonstrated in contact-dependent growth assays (Figure 1A). To overcome JQ1 resistance and in search of possible druggable interaction partners of BRD4, we first intended to better understand the possible mechanism of action in EwS. By the use of chromatin immunoprecipitation (ChIP) experiments, we observed that BRD4 interacts with the EWSR1 promoter, providing a rationale for the previously observed inhibition of the EWS-FLI1 mediated transcription program by JQ1 (Figure S1A, [18]).

Furthermore, previous publications indicated that BRD4 regulates polymerase II transcription by recruiting the positive transcription elongation factor P-TEFb and the concomitant stimulation of its kinase activity for phosphorylation of the C-terminal domain (CTD) of RNA polymerase II [34]. Subsequently, it was observed that anti-apoptotic genes were induced via P-TEFb, especially through its subunit CDK9 [29,35]. To study their possible involvement in the context of EwS, co-immunoprecipitation (Co-IP) studies were carried out to analyze whether BRD4 and pCDK9 interact with each other and whether there is also an interaction with EWS-FLI1. EwS cell lines A673, SKNMC and TC-71, were selected and analyzed after Co-IP with antibodies detecting BRD4, EWS-FLI1, and pCDK9 (Figure 1B). Indeed, co-elution of both phosphorylated CDK9 (p-CDK9) as well as EWS-FLI1 was observed after the BRD4 pull-down and not in the negative IgG control (Figure 1B). However, densitometry of repeated Western blotting of CoIPs demonstrated only enrichment of p-CDK9 in the precipitate although EWS-FLI1 was clearly present (Figure S1B). Enrichment of p-CDK9 is in agreement with previous observations [21,22,36], providing a rationale to investigate the potential of CDK9 inhibitors. 

CDK9 is widely expressed in different pediatric sarcomas (Figure 1C) and other pediatric and adult tumor entities (Figure S1C), while the local tumor site in EwS may influence its level of expression (Figure 1D). Interestingly, we observed that the level of CDK9 expression may be associated with event-free survival in EwS (Figure 1E). Impaired survival associated with low CDK9 expression, may be correlated with increased MCL1 expression (see Figure S1D) since increased MCL1 expression has been demonstrated to be associated with CDK9 drug resistance in leukemia [37], although this has to be further evaluated in a larger cohort of patients.

### 2.2. CDK9 Inhibitors Block EWS-FLI1 Expression and Proliferation of EwS

**Figure 2:**
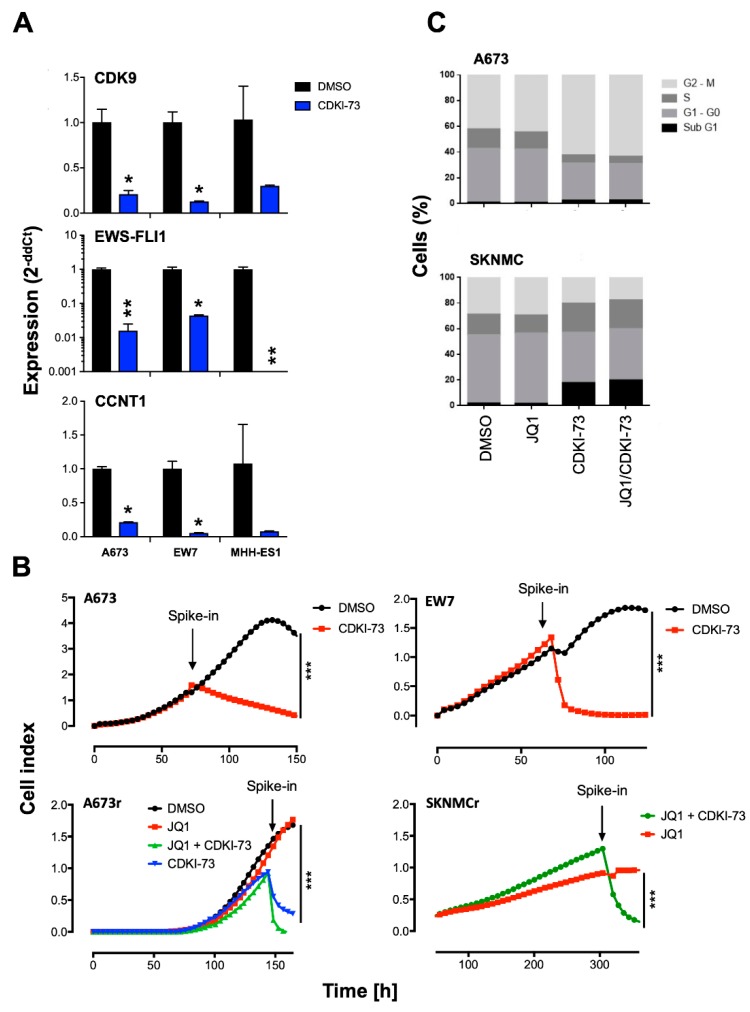
Effects of a new CDK9 inhibitor CDKI-73 on expression, proliferation, and cell cycle. (**A**) Analysis of expression of CDK9, CCNT1, and EWS-FLI1 24 h after treatment with 2 µM CDKI-73 in A673, EW7, and MHH-ES1 cell lines measured by qRT-PCR. Data are mean ± SEM; *t*-test. (**B**) Proliferation of EwS WT cell lines A673 and EW7 or JQ1-resistant A673 (A673r) and SKNMC (SKNMCr) cells was analyzed by xCELLigence assay. Upon reaching exponential growth rate, cells were treated with 2 µM CDKI-73/JQ1 or DMSO as negative control. Cellular impedance was measured every 4 h (relative cell index). Data are mean ± SEM (hexaplicates/group); *** *p*-value < 0.0005; 2-way ANOVA. (**C**) Cell cycle was analyzed 24 h after treatment with 1 µM JQ1, 1 µM CDKI-73, or both (each 1 µM) by flow cytometry of A673 and SKNMC. Cells were stained with propidium iodide. ** *p*-value < 0.005, * *p*-value < 0.05.

Since we found the interaction of BRD4 and p-CDK9 proteins, we further asked whether CDK9 inhibition may cause similar and/or additional effects compared to BRD4 inhibition in EwS. To analyze this hypothesis, we evaluated a new CDK9 inhibitor [38] in the EwS cell lines A673, EW7 (both possessing a type 1 EWS-FLI1 translocation), and MHH-ES1 (type 2 EWS-FLI1 translocation) (Figure 2A). Interestingly, qRT-PCR for CDK9 but also for EWS-FLI1 and cyclin T1 (CCNT1) revealed a significant decrease of expression especially after treatment with CDKI-73 for 24 h (Figure 2A). Furthermore, even though BRD4 expression was also reduced after CDKI-73 treatment, it was less suppressed compared to CDK9, CCNT1, or EWS-FLI1 (Figure S2A). Next, we analyzed whether the observed strong expression changes also influence cell proliferation. The xCELLigence assay was used for A673, EW7, and TC-71 cell lines. Cells were seeded and grown until they reached exponential growth phase. Cells were treated with 2 µM CDKI-73 or solvent control and monitored until growth regressed in the control. Consistent among all cell lines tested, treatment with CDKI-73 repressed cell growth most significantly in EW7 and TC-71 (Figure 2B and Figure S2B). For A673 cells, we also observed a block of proliferation that was as strong as observed for the other cell lines. However, treatment of JQ1 resistant cells with CDKI-73 revealed that these cells were still sensitive to CDK9i and confirmed a BET bromodomain independent mechanism in EwS (Figure 2B, A673r, SKNMCr). 

Subsequently, we investigated if the reduced proliferative activity of EwS cell lines after treatment with CDK9i may also influence cell cycle progression. To do this, EwS cell lines were treated for 24 h with CDKI-73 or JQ1 alone or both in combination. Flow cytometry analysis of propidium iodide stained cells demonstrated that CDKI-73 significantly reduced the cell population at the G1 phase of the cell cycle in most cases while simultaneously increasing apoptotic cells at the sub-G1 phase (Figure 2C and Figure S2C). Furthermore, results in SKNMC and TC-71 indicated a reduced G2/M phase with more cells in the S phase, as observed for SKNMC (Figure 2C and Figure S2C). Overall, the combination of CDKI-73 with JQ1 at the concentrations used did not further increase single CDKI-73 effects on the cell cycle (Figure 2C).

### 2.3. Expression Profile of EwS Cells after Treatment with CDK9 Inhibitors

**Figure 3:**
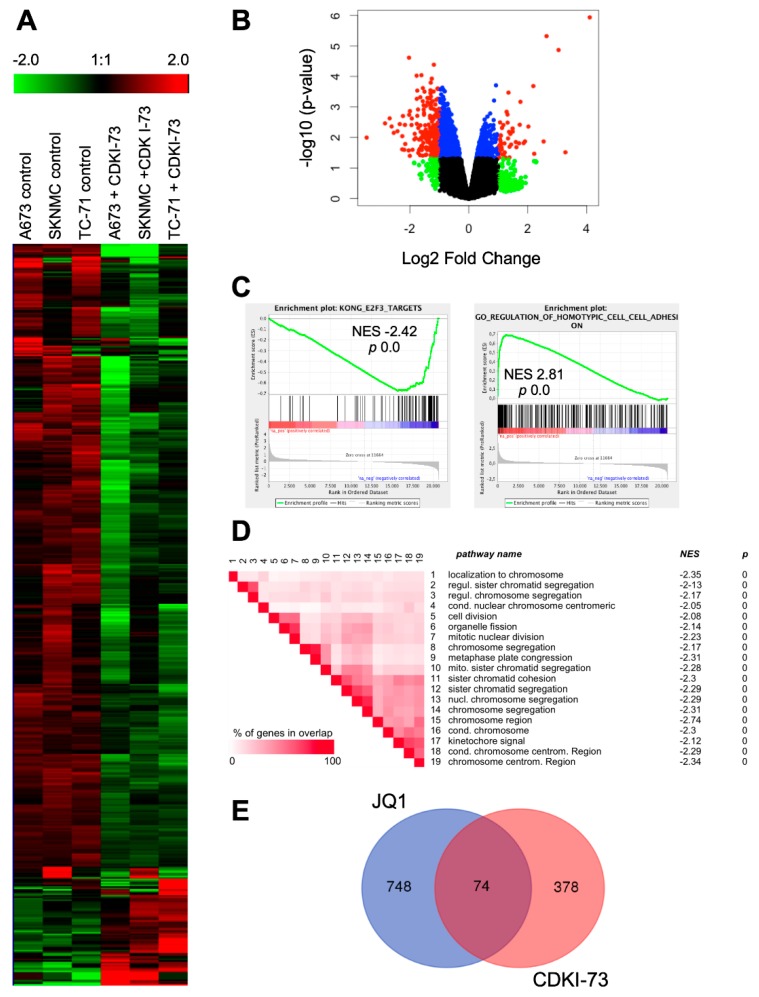
Expression profile of EwS cells after treatment with CDKI-73. (**A**) Heat map of 504 genes, 1.5-fold differentially expressed in 3 different EwS lines A673, SKNMC, and TC-71 are shown. Each column represents 1 individual array. Microarray data with their normalized fluorescent signal intensities were used (robust multichip average (RMA), see Materials and Methods; GSE119546). Cells were treated with DMSO or CDKI-73 for 8 h, collected, and then analyzed. (**B**) Volcano plot for CDKI-73 treated against solvent control EwS cell lines (A673, SKNMC, TC-71), showing the significance *p*-value (−log _10_) plotted over fold change (log 2). The color coding is as follows: red, genes with a *p*-value <0.05 and an absolute log FC > 1; blue, genes obtaining a *p*-value <0.05 and an absolute log FC ≤1; green, genes with a *p*-value ≥0.05 and an absolute log FC >1; and black, genes with a *p*-value ≥0.05 and an absolute log FC ≤1. (**C**) Gene set enrichment analysis (GSEA) enrichment plots of down- and up-regulated gene sets after CDKI-73 treatment. NES: normalized enrichment score. GSEA: http://www.broadinstitute.org/gsea/index.jsp (**D**) GSEA leading-edge analysis of identified gene sets up- or down-regulated after CDKI-73 treatment (C5_all, GO gene sets), respectively. Set-to-set analysis shows a correlation between CDKI-73 treatment and down-regulation of gene sets important for chromosomal regulation. (**E**) Shared genes differentially expressed after either CDKI-73 or JQ1 treatment in different EwS lines (see [18]; GSE72673), respectively. For Venn diagram, genes ± 1.5-fold differentially expressed were selected for the analysis (http://bioinformatics.psb.ugent.be/webtools/Venn/).

To better understand the CDK9i-induced changes in gene expression, EwS cells A673, SKNMC and TC-71 were treated with 2 µM CDKI-73 or solvent control for 8 h, RNA was isolated and hybridized onto Human Gene 1.0 ST arrays (Affymetrix). Analysis of differentially expressed genes at a fold change of ±1.5 revealed 74 up- and 431 down-regulated genes following CDKI-73 treatment (Figure 3A). Differentially expressed genes using volcano plots indicated 49 and 254 genes significantly up- and down-regulated upon treatment over all cells analyzed, respectively (*p*-value <0.05; Figure 3B). Subsequent gene set enrichment analysis (GSEA) identified down-regulation of typical E2F3 target gene sets as identified by Kong et al. (2007) [39] as well as an up-regulation of genes associated with cell adhesion, possibly counteracting invasive tumor growth (Figure 3C). In addition, GSEA leading-edge analysis identified a number of gene sets down-regulated after CDKI-73 treatment that seem to be important for chromosomal regulation of mitosis (Figure 3D), providing a potential rationale for the observed disturbed G2/M phase of the cell cycle in some EwS cell lines. Subsequently, we compared the de-regulated transcriptome of EwS cell lines after CDKI-73 treatment to that of genes differentially expressed after the JQ1 exposition (see Hensel et al. (2016) [18]) by Venn analysis (http://bioinformatics.psb.ugent.be/webtools/Venn/). Although our and previously published [22] data indicate that BRD4 and CDK9 localize together and regulate transcription in EwS, we observed only 74 genes simultaneously deregulated by either CDKI-73 or JQ1 treatment (Figure 3E), respectively. This together with differential GSEA data (Figure 3C,D and Hensel et al. (2016) [18]) may indicate that BRD4 and p-CDK9 are not always in a complex together or are associated with different cofactors at different gene loci to modulate gene expression.

A focused expression array analysis of genes involved in the regulation of transcription differentially regulated after inhibition of CDK9 activity in EwS cell lines identified 59 genes down-regulated and only 5 genes up-regulated (Figure S2D). Interestingly, the most significant genes up-regulated included genes of the AP-1 family of transcription factors such as *FOS* and *JUNB,* while down-regulated genes included *E2EF3*, EwS specific transcription factors such as *NR0B1,* but also genes involved in epigenetic regulation such as *SUV39H1*. Their differential expression was independently verified by qRT-PCR (Figure S2E).

In the next step, to evaluate heterogenicity and possible differential responsiveness to CDKI-73 treatment in these three EwS cell lines, we investigated heterogeneity of gene ontology (GO) of deregulated biological processes. Venn diagram analysis demonstrated that only a limited number of deregulated genes are shared by all three cell lines (Figure S3A). Therefore, common deregulated biological processes can be identified (Figure S3B), but response heterogenicity to this drug seems significant (Figure S3C).

We further analyzed whether apoptotic mechanisms become activated after CDK9 inhibition, as previously observed after JQ1 treatment of EwS [18] (Figure S4A). EwS cell lines A673 and SKNMC were treated with 2 µM CDKI-73 for 2, 4, and 6 h, RNA was isolated and subsequently analyzed by qRT-PCR. We observed down-regulation in a time-dependent fashion not only of EWS-FLI1 but also of genes previously demonstrated to be strongly down-regulated after JQ1 treatment (Figure S4B).

### 2.4. Combined Targeting of CDK9 and BRD4 Inhibits Proliferation and Induces Apoptosis

**Figure 4:**
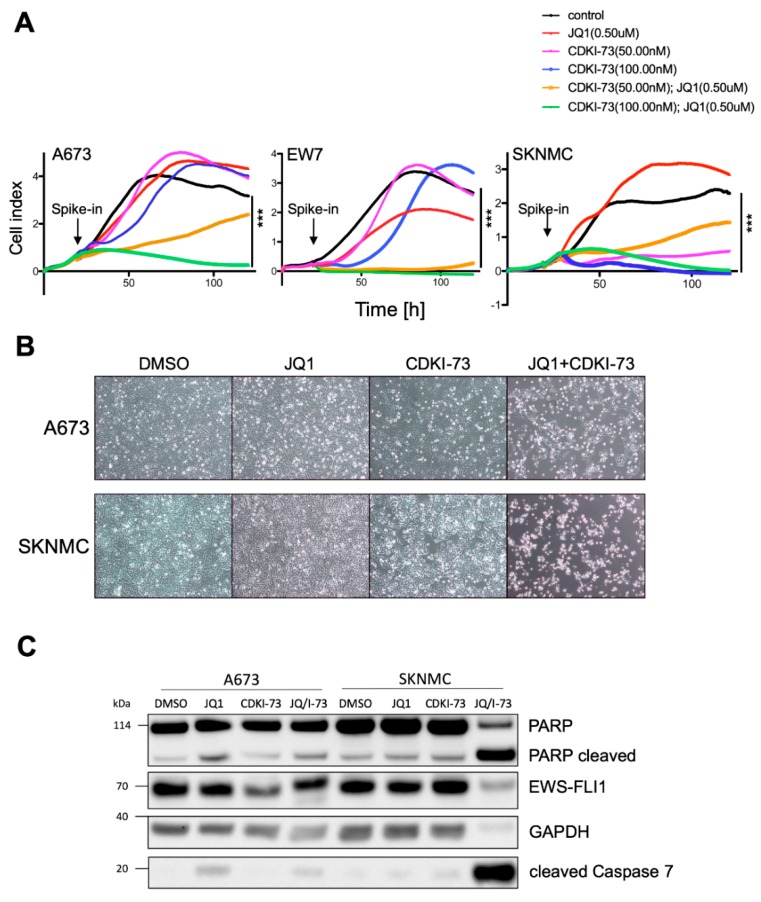
Combined targeting of CDK9 and BRD4 results in the inhibition of proliferation and induction of apoptosis. (**A**) Anti-proliferative activity of JQ1 and/or CDKI-73 against A673, EW7 and SKNMC cells by xCELLigence assay measuring cellular impedance every 4 h (relative cell index). Data are mean ± SEM (hexaplicates/group); 1-way ANOVA; *** *p*-value <0.0005. (**B**) Phase contrast microscopy (magnification 10×) of cells 48 h after treatment with inhibitors. A673 or SKNMC cells were treated for 48 h with either 500 nM JQ1, 100 nM CDKI-73, or both, compared to DMSO control. (**C**) Western blot analysis of apoptosis susceptibility after JQ1 and/or CDKI-73 (I-73) treatment, respectively. Protein levels measured by antibodies against EWS-FLI1, PARP, CASP7, and GAPDH as loading control. A673 or SKNMC cells were treated for 24 h with inhibitors as shown.

To further investigate combinatorial efficacy of JQ1 and CDKI-73, we first carefully determined IC50 concentrations of CDKI-73, which revealed an IC50 of 105.9 ± 44.7 nM on EwS cell lines (Figure S4C). Subsequent combined treatment with low doses of JQ1 and/or CDKI-73 in contact dependent proliferation assays of A673, EW7, and SKNMC cells demonstrated that single low doses in most cases were not effective while their combination revealed a strong synergism to suppress cellular proliferation (Figure 4A). In SKNMC cells, CDKI-73 treatment alone was already effective at 50 nM concentration, but the combination with JQ1 was more effective at a 100 nM concentration of CDKI-73. Combined low dose efficacy of both drugs was also obvious in morphology (Figure 4B). Furthermore, Western blot analysis of cleaved caspase 7 and PARP expression in EwS cell lines treated with low doses of JQ1 and/or CDKI-73 for 24 or 48 h demonstrated a strong induction of apoptosis when cells received combined treatment, with low doses of JQ1 already effective in A673 cells (Figure 4C and Figure S4D).

### 2.5. Combined Targeting of CDK9 and BRD4 Inhibits Tumor Growth

To further investigate whether CDKI-73 might be of therapeutic value in vivo, we combined low dose JQ1 and CDKI-73 treatment and compared it to vehicle as well as single agent treatment. To do this, a xenograft mouse model of Rag2^−/−^γ_c_^−/−^ mice was used and 3 × 10^6^ A673 cells were injected subcutaneously (s.c.). As soon as tumors were palpable, mice were randomly divided into four groups and treatment commenced. Animals received a dosage of 50 mg/kg body weight JQ1 or corresponding vehicle every 24 h per i.p. injection. CDKI-73 or corresponding vehicle was administered via oral gavage at 50 mg/kg body weight every 48 h. Treatment was carried out for a maximum of 14 days. Tumor size was measured daily until the tumor exceeded 1 cm^3^ in size whereupon mice were sacrificed. 

**Figure 5:**
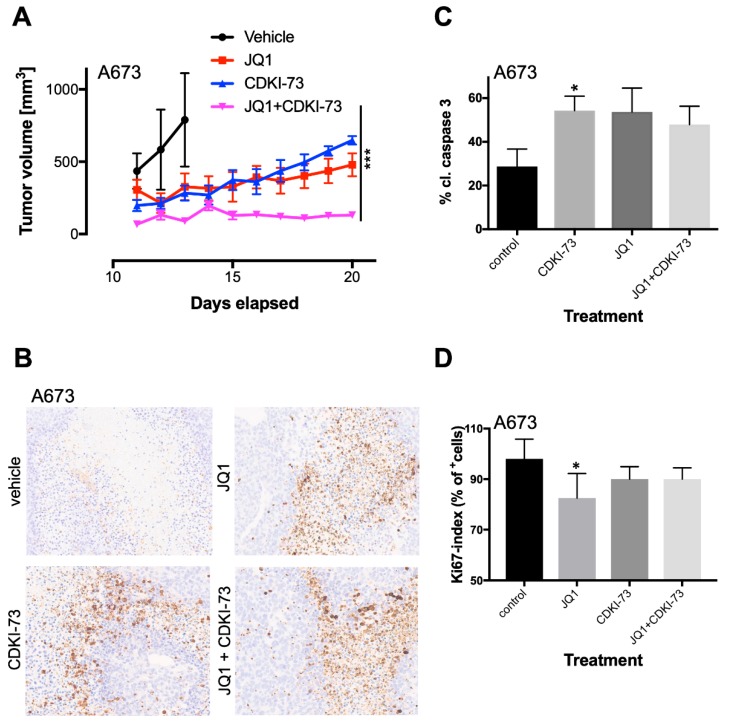
Combined targeting of CDK9 and BRD4 results in the inhibition of proliferation and tumor growth. (**A**) In vivo anti-tumor efficacy of JQ1 and CDKI-73 as a single agent or in combination against A673-bearing Rag2^−/−^γ_C_^−/−^ mice xenografts. A673 cells were injected subcutaneously (s.c.) into mice. Once tumor was palpable mice were randomly allocated into four groups (7 mice/group) and treated with vehicle (DMSO), JQ1 (50 mg/kg, i.p., daily), CDKI-73 (50 mg/kg, p.o. every other day), or with CDKI-73 in combination with JQ1. For the combination study, JQ1 was given i.p. daily, followed by CDKI-73 every other day. Two-way ANOVA; *** *p*-value <0.0005. (**B**) As soon as tumors reached 1 cm^3^, mice were sacrificed and tumors were analyzed for cleaved caspase 3 by immunohistochemistry (see Materials and Methods). Representative results of A673 tumors are shown (4× original magnification). (**C**) The level of caspase 3 in A673 tumors. The percentage of cleaved caspase 3 positive cells in five fields per tumor is given; *t*-test, * *p*-value <0.05. (**D**) The percentage of Ki67 positive cells in five fields per tumor is given; *t*-test, * *p*-value <0.05.

Analysis of tumor growth between different groups over time of treatment revealed the lowest tumor growth rate for combination treatment (Figure 5A). Interestingly, this combination treatment was similar in efficacy, as observed after high dosage JQ1 treatment demonstrated previously [18]. Single-agent treatment of JQ1 or CDKI-73 also delayed tumor growth, although they finally caught up to tumor size in the control group. However, identical treatment of SKNMC cells injected s.c. (6 mice/group) did not show the same synergism as observed with A673 cells (Figure S5A), which could be due to overall heterogeneity of biological processes deregulated in individual EwS lines by this drug, as observed by array analysis (see above). It could also be the result of a compromised RB function that was recently identified [40] and only became apparent at low doses of CDK9i treatment. Immunohistochemistry of A673 tumors demonstrated an increased cleaved caspase 3 expression in treated tumors compared to vehicle treatment, but no significant differences were observed within treatment groups (Figure 5B,C). Furthermore, Ki67 analysis of tumor histology demonstrated reduced proliferation in all treatment groups that was not increased following combined treatment (Figure 5D). However, Ki67 analysis of combined JQ1/CDKI-73 treatment in SKNMC tumors revealed increased proliferation when compared to control tumors (Figure S5D). In SKNMC tumors, CDKI-73 induced the highest level of cleaved caspase 3 compared to the treatment with either JQ1 or its combination with CDKI-73 (Figure S5B,C), suggesting additional mechanisms influencing tumor growth.

## 3. Discussion

The pathognomonic EWS-FLI1 transcription factor uses divergent chromatin remodeling mechanisms to activate or repress transcription in Ewing sarcoma (EwS) [10,11,41]. It induces altered epigenetic marks generating specific acetyl-lysine moieties on histone H3 that are targeted by epigenetic reader proteins such as BRD4 [18]. Inhibition of BRD4 by JQ1, dBET, or iBET significantly blocked proliferation and in vivo tumor growth of different EwS lines and strikingly resulted in a strong down-regulation of the EWS-FLI1 mediated transcription program [18] (Becker-Dettling, data not shown). According to other reports [33], we also observed development of resistance following long term treatment of EwS cells with JQ1. 

Transcriptional initiation and elongation are complex processes involving an array of proteins and regulated steps. By binding of BRD4 to acetylated lysines at discrete chromosomal locations, it recruits further regulatory complexes to promote gene expression [42]. BRD4 controls the recruitment of P-TEFb [22] that regulates the release of promoter-proximal pausing genome-wide and consists of the kinase CDK9 and Cyclin T1 [20]. In EwS, an interaction of BRD4 with EWS-FLI1 was demonstrated [43]. We also observed a direct binding of BRD4 to the *EWS-FLI1* promoter. Furthermore, by Co-IP experiments, we demonstrated a direct interaction of BRD4 with phosphorylated CDK9 in EwS, as previously shown in other tumor entities.

This prompted us to investigate the functional contribution of CDK9 to transcriptional elongation in EwS. We used inhibitors of CDK9 that had already demonstrated a higher selectivity for CDK9 than previously used compounds such as Flavopiridol [35] and have the advantage of being orally bioavailable. CDKI-73 has been described as a new promising CDK9 inhibitor for several tumor entities [35,38,44,45]. In EwS, CDKI-73 reversed resistance to JQ1 and the combined treatment of CDKI-73 and JQ1 complemented their inhibitory activity and enabled dose reduction in vitro and in vivo.

To better understand the inhibitory activity of CDKI-73, we analyzed the transcriptional profile of EwS cells after treatment with CDK9i. Although both CDKI-73 and JQ1 treatment resulted in the suppression of an EwS specific expression profile, Venn diagram analysis demonstrated that only a minor fraction of genes is co-targeted by both drugs. This indicates that although BRD4 and p-CDK9 are identified to co-exist in an active transcriptional complex [22], confirmed also in EwS, temporal interaction with other proteins involved in transcriptional elongation may significantly contribute to their different profiles. There is also evidence that BRD4 is crucial for productive transcriptional elongation but not for direct recruitment of P-TEFb [46]. Furthermore, a recent publication indicates that genes including tumor suppressor genes may be selectively suppressed via P-TEFb activity [27].

Active P-TEFb was identified by GSEA to activate E2F3 target genes in EwS necessary for the G1 to S phase transition [39] affecting DNA replication and mitotic activity, which was further validated by GSEA leading-edge analysis providing a potential rationale for the observed disturbed G2/M phase of EwS cells following CDK9 inhibition. Moreover, CDKI-73 also downregulated EwS typical transcription factors such as *NR0B1* required for oncogenic phenotype [47] and strongly induced AP-1 family of transcription factors such as *FOS* and *JUNB* that are presumably important for cellular differentiation of EwS.

Active P-TEFb like BRD4 induces the expression of anti-apoptotic genes such as *CFLAR* and *XIAP* [18], and CDKI-73 promotes cleaved caspase 7 and PARP in EwS cell lines. However, CDKI-73 does not further increase apoptosis of cells already treated with JQ1 and vice versa, indicating the induction of similar apoptotic mechanisms in EwS [18].

CDKI-73 has shown a synergistic effect with JQ1 [18], as demonstrated by inhibiting cellular proliferation and tumor growth in vivo, at least for A673 cells. The diminished synergistic activity against SKNMC cells in vivo might be related to known functional mutations in the RB pathway [40] or overall functional heterogenicity, as demonstrated by GO analysis presumably affecting the efficacy of CDK9 inhibition.

In transcriptionally driven tumors such as EwS, the functional interaction of BRD4 with P-TEFb is therapeutically interesting. Targeting both BRD4 and CDK9 complements affected functional differences of genes identified in EwS; thus, co-targeting BRDs and P-TEFb can be developed as an effective strategy for preventing or overcoming the drug resistance to JQ1. We here demonstrate that co-treatment of the EwS-bearing Rag2^−/−^γ_C_^−/−^ mice with low doses of CDKI-73 and JQ1 markedly inhibited tumor growth when compared to the treatment with vehicle or with either drug alone, whereby therapeutic efficacious doses were lower for JQ1 than previously demonstrated [18]. 

## 4. Materials and Methods 

### 4.1. Cell Lines 

EwS lines (MHH-ES1, SKNMC, and TC-71), were obtained from the German Collection of Microorganisms and Cell Cultures (DSMZ, Braunschweig, Germany). A673 was purchased from ATCC (LGC Standards, Teddington, UK). EW7 cells were kindly provided by O. Delattre (Institut Curie, Paris, France). Cells were maintained in a humidified incubator at 37 °C in 5% CO_2_ atmosphere in RPMI 1640 (Life Technologies, Darmstadt, Germany) containing 10% heat-inactivated fetal bovine serum (Life Technologies) and antibiotics (Life Technologies). Cell lines were routinely checked for purity (e.g., EWS-FLI1 translocation product, surface antigen, or HLA-phenotype) and mycoplasma contamination as well as for identity by DNA profiling using 8 different and highly polymorphic short tandem repeat (STR) loci (DSMZ, Braunschweig, Germany).

### 4.2. Chemical Compounds

The synthesis of the CDK9 inhibitor was described previously [38,48]. The compound was dissolved in dimethylsulfoxide (DMSO at a stock concentration of 10 mM) and stored at −20 °C.

### 4.3. Proliferation Assay

Cell proliferation was measured with an impedance-based instrument system (xCELLigence, Roche/ACEA Biosciences, Basel, Switzerland), enabling label-free real-time cell analysis. Briefly, 2–4 × 10^4^ cells were seeded into 96-wells with 200 µL media containing 10% FBS and allowed to grow up to 150 h. Cellular impedance was measured periodically every 4 h and JQ1, CDK9 inhibitor, or DMSO was added as indicated in the results section.

### 4.4. Co-Immunoprecipitation (Co-IP)

For Co-IP, cells were seeded at 6–10 × 10^6^ per cell culture dish and cultivated until 90% confluence. Cells were subsequently harvested and treated using the Universal Magnetic Co-IP Kit (Active Motif, Carlsbad, CA, USA). Briefly, cells were washed twice with ice-cold PBS containing inhibitors. Whole cell lysis buffer containing inhibitors was added to the cell pellet and incubated on a rotating platform at 4 °C for 30 min. The cleared supernatant was supplemented with the precipitating antibody and added to prepared protein G magnetic beads incubated o/n at 4 °C and constant rotation. The beads-antibody-antigen conjugate was then washed three times using 500 μL complete Co-IP/wash buffer and the proteins eluted into Laemmli sample buffer and denatured for 10 min at 68 °C. Eluates were analyzed by Western blotting.

### 4.5. Western Blot Analysis 

Treatment of 5 × 10^5^ A673, SKNMC or TC-71 cells with 500 nM JQ1, 100 nM CDKI-73, or the combination of both; DMSO was used as solvent control. Cells were washed once with PBS and harvested in lysis buffer containing 50 mM Tris HCl, 10 mM beta-glycerophosphate, 150 mM NaCl, 1% Triton-X 100, 5 mM Na_4_PO_7_, 1 mM Na_3_VO_4_, 50 mM NaF supplemented with protease inhibitor (Complete mini, Roche Diagnostics). Protein concentration was determined by BCA (Thermo Fisher Scientific, Waltham, MA USA). Ten to thirty micrograms of protein extract were resolved on 4%–12% SDS-PAGE and transferred onto a nitrocellulose membrane (Thermo Fisher Scientific).

Primary antibodies were used as follows: anti-BRD4 rabbit monoclonal antibody (Abcam, Cambridge, USA), anti-FLI1 monoclonal antibody (Cell Signaling Technology, Danvers, MA, USA), anti-pCDK9 (Cell Signaling Technology), anti-PARP rabbit polyclonal antibody (Cell Signaling Technology), anti-Caspase7 antibody (Cell Signaling) and loading control GAPDH (Cell Signaling Technology). After incubation with the appropriate secondary peroxidase-conjugated antibodies, detection was performed with the ECL chemiluminescence reagent (Amersham Biosciences, Little Chalfont, UK) or SuperSignal West Femto (Thermo Fisher Scientific). 

### 4.6. Cell Cycle Analysis

Treated cells were washed with PBS, collected, fixed with 70% ethanol for 2 h on ice and stained with PI solution (20 µg/mL PI; Sigma-Aldrich, St. Louis, MO, USA) for 1 h at room temperature in PBS, 0.1% Triton X, 200 µg/mL RNAse A. Cells were measured on a FACS Canto. Data were analyzed using Flow Jo program (Flow Jo LLC., Ashland, OR, USA).

### 4.7. RNA Isolation

RNA from frozen tissue or cultured cells was isolated using the TRI Reagent RNA Isolation Kit (Thermo Fisher Scientific). Briefly, up to 1 × 10^7^ cells were resuspended in TRI Reagent solution and either used immediately or stored at −80 °C for further use. Lysed cells were then mixed with 100 µL BCP (per ml TRI Reagent) by vortexing and incubated on ice for 5–15 min. Subsequently phases were separated by centrifugation (12,000 × *g*) for 5 min at 4 °C. The aqueous phase containing RNA was then separated and mixed with 500 µL isopropanol by vortexing. After an incubation for 10 min on ice, precipitated RNA was centrifuged again at 4 °C and 12,000 × *g* for 8 min. The RNA was then washed with 1 ml 70% ethanol, centrifuged at 7,500 × *g* and 4 °C for 5 min and briefly air dried. The remaining RNA pellet was resuspended in DEPC-treated H2O. RNA concentration was measured at 260 nm using a nanophotometer (Implen, Munich, Germany) and stored at −80 °C.

### 4.8. Quantitative Real Time-PCR (qRT-PCR)

Total RNA was isolated and reverse transcribed using the High Capacity cDNA Reverse Transcription Kit (Thermo Fisher Scientific) according to the manufacturer’s instructions. Differential gene expression was then analyzed by qRT-PCR using TaqMan Universal PCR Master Mix and fluorescence detection with a Step One Plus Real-Time PCR or ABI 7900 instrument (Thermo Fisher Scientific), as described previously [49,50]. Gene expression was normalized to glyceraldehyde-3-phosphate dehydrogenase (GAPDH) using the ΔΔCT method [51]. All experiments were performed at least in duplicate for each cell line. A list of assays used is provided in the supplementary information.

### 4.9. Microarray Analysis

Biotinylated target cRNA was prepared as previously described [49]. A detailed protocol is available at www.affymetrix.com. Samples were hybridized to Affymetrix Human Gene 1.0 ST microarrays and analyzed by an Affymetrix software expression console, version 1.1. For the data analysis, robust multichip average (RMA) normalization was performed, including background correlation, quantile normalization, and median polish summary method. The volcano plot was generated using R (version 3.4.4; https://www.R-project.org/). To calculate *p*-values and log2 fold changes, the genes were tested for differential expression between groups based on log2 expression values. Before testing, all probe set ids without mapped gene symbols were removed. The remaining 21946 genes were tested for differential expression using the moderated t-statistic of the R package limma with default settings. For gene ontology analysis, overlapping up-regulated or down-regulated genes were identified using the jvenn platform [52] and analyzed via the Metascape platform with standard settings for pathway enrichment using GO Biological Processes, KEGG Pathway, Canonical Pathway, and Reactome Gene Sets databases [53].

### 4.10. Animal Model

Immune deficient Rag2^−/−^γc^−/−^ mice on a BALB/c background were obtained from the Central Institute for Experimental Animals (Kawasaki, Japan) and maintained in our animal facility under pathogen-free conditions in accordance with institutional guidelines and approval by local authorities (55.2.1-54-5232-42-2016). Experiments were performed in 6–20-week old mice.

### 4.11. In Vivo Experiments

To examine in vivo tumorigenicity, 2–3 × 10^6^ EwS cells were injected subcutaneously into immune-deficient Rag2^−/−^γc^−/−^ mice. Once the tumor was palpable, mice were divided randomly into four groups and treated either with 50 mg/kg body weight JQ1, CDKI-73, or JQ1 in combination with CDKI-73, or solvent controls. JQ1 was handled and dissolved as recommended by the Bradner lab and administered at 50 mg/kg body weight intra peritoneal (i.p.) daily while CDKI-73 was administered by oral gavage every 48 h. Mice were monitored daily and tumor xenografts were measured with digital calipers, and tumor volume was calculated as (L × W^2^)/2, where L is length and W is width. Experimental endpoints were determined by completion of treatment or attainment of tumor burden exceeding 1 cm^3^. Upon reaching endpoints, mice were humanely euthanized, tumors excised and characterized. 

### 4.12. Histology

Histological analysis of tumor specimens was performed in 5 tumors per group. Tissues were fixed in 10% neutral-buffered formalin solution for minimum 48 h, dehydrated under standard conditions (Leica ASP300S, Wetzlar, Germany) and embedded in paraffin. Serial 2 µm-thin sections prepared with a rotary microtome (HM355S, Thermo Fisher Scientific) were collected and subjected to histological and immunohistochemical analysis. Hematoxylin–Eosin (H&E) staining was performed on deparaffinized sections with Eosin and Mayer’s Haemalaun, according to a standard protocol. Immunohistochemistry was performed using a Bond RXm system (Leica, Wetzlar, Germany, all reagents from Leica) with primary antibodies against cleaved caspase 3 (clone ASP175, 1:150, Cell Signaling 9664) for the detection of apoptosis and Ki67 (clone: SP6, 1:50, ab16667) for cell proliferation. Slides were deparaffinized and pretreated with Epitope retrieval solution 1 (citrate buffer pH6). The primary antibody was diluted as described above and applied for 15 min. Antibody binding was detected with a polymer refine detection kit without post primary reagent and visualized with DAB as a dark brown precipitate. Counterstaining was done with hematoxyline. Slides were then dehydrated manually by alcohol washes of increasing concentration (70%, 96%, 100%) and xylene and cover slipped using Pertex^®^ mounting medium (Histolab, Goeteborg, Sweden, 00801). A positive control was included in each run.

All slides were scanned with a high-throughput scanning system (AT2, Leica). The percentage of tumor area occupied by cleaved caspase 3 positive tumor cells and the percentage of Ki67 positive tumor cells was evaluated in all sections by experienced pathologists (cleaved caspase: HYY /KS; Ki67: KS), and representative images were taken using Imagescope (version 12.4.7018, Leica).

### 4.13. Statistical Analyses

Data are mean ± SEM as indicated. Differences were analyzed by unpaired two-tailed Student’s *t*-test, as indicated using Excel (Microsoft, Redmond, WA, USA) or Prism 7 (GraphPad Software, San Diego, CA, USA) as well as by 1-way and 2-way analysis of variance (ANOVA) using Prism 7; *p*-values <0.05 were considered statistically significant (* *p* < 0.05; ** *p* < 0.005; *** *p* < 0.0005). Volcano plots were drawn using R, a free software environment available at http://www.r-project.org/. 

## 5. Conclusions

Translocation driven tumors such as EwS are very sensitive to combined treatment with inhibitors targeting transcriptional elongation. Overall, our results show that the EWS-FLI1 mediated pathognomonic expression programs may be similarly targeted by BET bromodomain and CDK9 inhibition, providing a rationale for future combination therapy of this disease.

## Supplementary Materials

The following are available online at https://www.mdpi.com/2072-6694/12/2/304/s1, Supplementary information; Figure S1: The BRD4, CDK9 expression axis and its influence on cell cycle and proliferation; Figure S2: Influence of CDK9 inhibition on transcriptional regulation and cell cycle progression, Figure S3: GO Analysis of differentially regulated genes, Figure S4: Influence of CDK9 inhibition on the regulation of apoptosis, Figure S5: Combined targeting of CDK9 and BRD4 of SKNMC tumors results in the inhibition of proliferation and tumor growth, Figure S6: Whole Western blots with molecular weight markers and densitometry of individual Western blot figures, Figure S7: Western blots used for densitometry in Figure S1B.
