# Supplementary material for: Combined Inhibition of Epigenetic Readers and Transcription Initiation Targets the EWS-ETS Transcriptional Program in Ewing Sarcoma
Source: Cancers (Basel). 2020 Jan 28;12(2):304. doi: 10.3390/cancers12020304 (PMC7072515; doi:10.3390/cancers12020304)
Supplement: Supplementary file 1 [file cancers-12-00304-s001.pdf]

Supplementary Information

Materials and Methods

Chromatin Immunoprecipitation (ChIP)

All steps were carried out at 4 °C unless otherwise noted. Cells were seeded at 6–10 × 10^6 and cultivated for 24–72 h at 37 °C. Subsequently, crosslinking was done by incubation with formaldehyde (Life Technologies) at a final concentration of 1% for 10 min and 37 °C, followed by quenching with 0.125 M glycine for 5 min and 37 °C. Cells were lysed in lysis buffer 1–3 (see below) for 10 min each. The released chromatin was then sonicated to an average DNA length of 200–600 bp using a Bioruptor Pico (Diagenode, Seraing, Belgium). Chromatin immunoprecipitation (ChIP) was carried out using 5 µg of anti-BRD4 antibody (EPR5150(2); Abcam, Cambridge, UK) or rabbit IgG (sc-2027X; Santa Cruz, Heidelberg, Germany), respectively. Beads for IP were also incubated overnight with 1 mg/mL BSA and cleared with wash buffer 1 (Additional file: Supplementary Materials and Methods) for 5 min. Chromatin-antibody conjugates were then incubated with pre-blocked beads for 3 hrs. Conjugates were washed with wash buffer 1–3 for 5 min each. Chromatin was eluted in elution buffer (Additional file: Supplementary Materials and Methods) at 65 °C for 15 min. Reverse crosslinking was performed by the addition of ribonuclease A (final concentration 200 µg/mL) and incubated for 45 min at 37 °C. Proteins were removed overnight (o/n) by the addition of 200 mM NaCl and proteinase K (200 µg/mL) at 65 °C. DNA was purified with QIAquick PCR purification Kit (Qiagen, Chatsworth, CA, USA). DNA concentration was measured with the Qubit® DNA HS Kit (Thermo Fisher Scientific, Braunschweig, Germany) on a Celigo® (Nexcelom, Lawrence, MA, USA) and quality was assessed on the 2100 Bioanalyzer (Agilent, Waldbronn, Germany) with the Agilent High Sensitivity Kit. Specificity was analyzed by genomic-PCR with specific promoter primers EWS-forward 5′-TAGTTACCCACCCAAACTGGAT-3′ and EWS-reverse 5′-GGGCCGTTGCTCTGTATTCTTAC-3′, and irrelevant control primers forward 5′-GGGCAGGAAGAGGGCCTAT-3′ and reverse 5′-GAGACGTGCTACTTCCATTTGTC-3′. BRD4 binding was normalized to IgG control antibody using the ΔΔCT method.

Buffer used for ChIP

Name	Ingredients
**11% Formaldehyde solution**	50 mM HEPES-KOH pH 7.5, 100 mM NaCl, 1 mM EDTA, 0.5 mM EGTA, 11% Formaldehyde
**Lysis buffer 1**	50 mM HEPES-KOH pH 7.5, 140 mM NaCl, 1 mM EDTA, 10% glycerol, 0.5% NP-40, 0.25% Triton X-100
**Lysis buffer 2**	10 mM Tris-HCL pH 8.0, 200 mM NaCl, 1 mM EDTA, 0.5 mM EGTA
**Lysis buffer 3**	10 mM Tris-HCL pH 8.0, 100 mM NaCl, 1 mM EDTA, 0.5 mM EGTA, 0.1% Na-Deoxycholate, 0.5% N-Lauroyl-sarcosine
**ChIP dilution buffer**	50 mM Tris-HCL pH8.0, 167 mM NaCl, 1.1% Triton X-100, 0.11% Na-Deoxycholate
**Wash buffer 1**	50 mM Tris-HCL pH 8.0, 0.1% SDS, 0.1% Na-Deoxycholate, 1% Triton X- 100, 150 mM NaCl, 1 mM EDTA, 0.5 mM EGTA
**Wash buffer 2**	50 mM Tris-HCL pH 8.0, 0.1% SDS, 0.1% Na-Deoxycholate, 1% Triton X- 100, 500 mM NaCl, 1 mM EDTA, 0.5 mM EGTA
**Wash buffer 3**	50 mM Tris-HCL pH 8.0, 250 mM LiCl, 0.5% Na-Deoxycholate, 0.5% NP-40, 1 mM EDTA, 0.5 mM EGTA

Primers and Assays Used for qRT-PCR

TaqMan Gene Expression Assays (Life Technologies) were used for the genes BRD4 (Hs04188087_m1), CCNT1 (Hs01059085_m1), CDK9 (Hs00977896_g1), CFLAR (Hs01116280_m1), E2F3 (Hs00605457_m1), FOS (Hs04194186_s1), GAPDH (Hs02758991_g1), HES1 (Hs00172878_m1), ID1 (Hs03676575_s1), JUNB (Hs00357891_s1), NR0B1 (Hs00230864_m1), SOX2 (Hs01053049_s1),

SUV39H1 (Hs00957892_m1), and XIAP (Hs00745222_S1). For EWS-FLI1 detection, the following primers 5′-TAGT-TACCCACCCCAAACTGGAT-3′ (sense), 5′-GGGCCGTTGCTCTGTATTCTTAC-3′ (antisense) and probe 5′-FAM CAGCTACGGGCAGCAGAACCCTTCTT-TAMRA-3′ were designed.

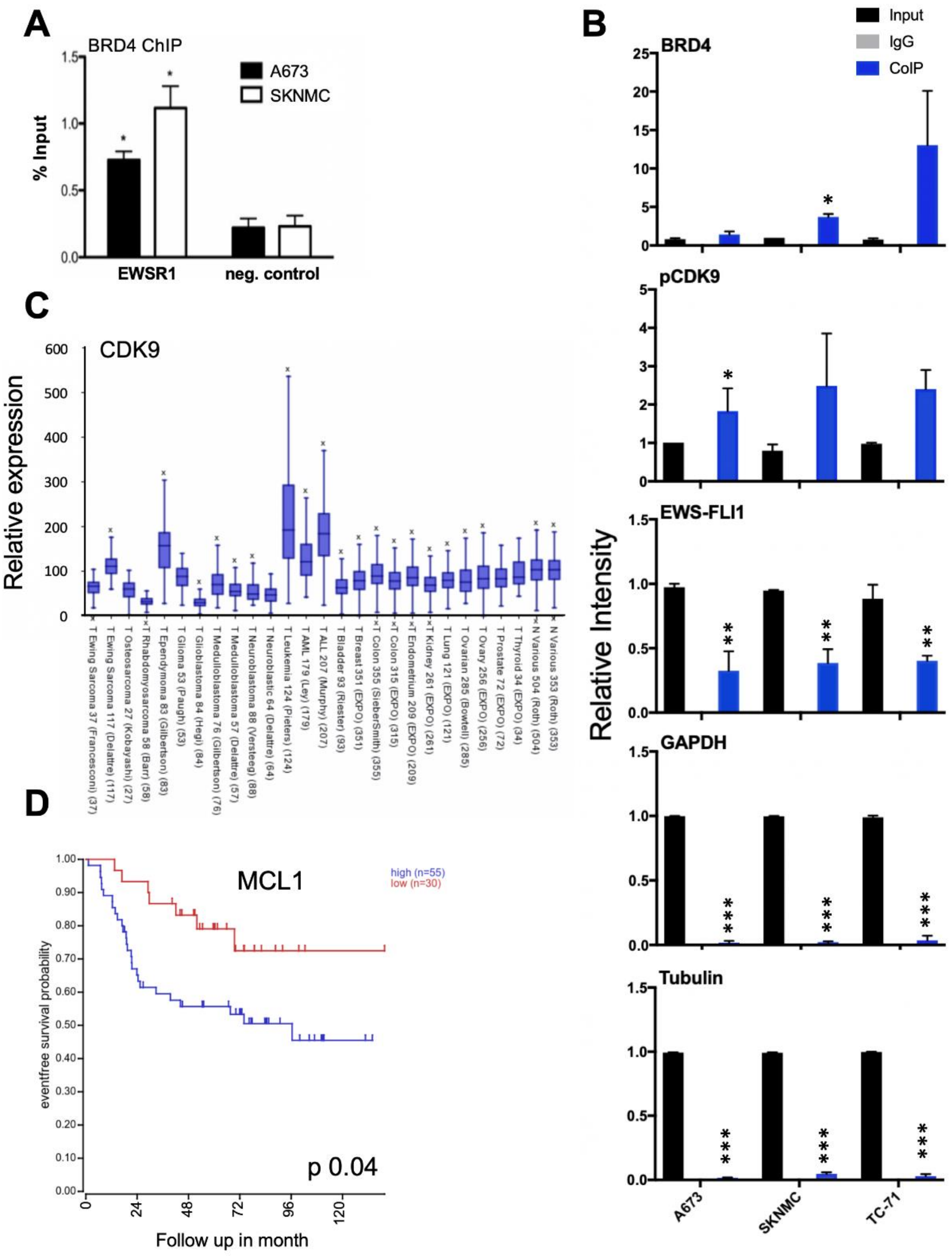

Supplementary Figure S1. The BRD4, CDK9 expression axis and its influence on cell cycle and proliferation. (**A**) Interaction analysis of BRD4 at the *EWSR1-FLI1* promoter by chromatin immunoprecipitation (ChIP). ChIP for BRD4 in A673 and SKNMC cell lines was done as described (see Materials and Methods). Retained DNA was analyzed by qRT-PCR using primer specific for the *EWSR1-FLI1* promoter (EWSR1) and compared to a region not enriched for BRD4 (negative control) *p*-value <0.05. (**B**) Densitometry of CoIP experiments to analyze interaction between BRD4, EWS-FLI1, and p-CDK9. Co-IP for A673, SKNMC and TC-71 cell lines using anti-BRD4 antibodies. After Co-IP, proteins were analyzed by Western blotting for p-CDK9, BRD4, and EWS-FLI1. GAPDH and Tubulin

served as a loading control. Cumulative densitometry of two Western blots per antibody is shown. Data are mean ± SEM; *t*-test *** *p*-value < 0.0005, ** *p*-value < 0.005, * *p*-value < 0.05. (**C**) Expression levels of CDK9 in different pediatric small-round-blue-cell tumors, carcinomas and normal tissues by box plot presentation using a comparative study of the amc onco-genomics software tool (https://hgserver1.amc.nl/cgi-bin/r2/main.cgi.). The number of samples in each cohort is given. (**D**) MCL1 expression correlates with event-free survival: Kaplan–Meier estimates for event-free survival probability for MCL1 expression (n = 85, p = 0.004), GSE63157 study set.

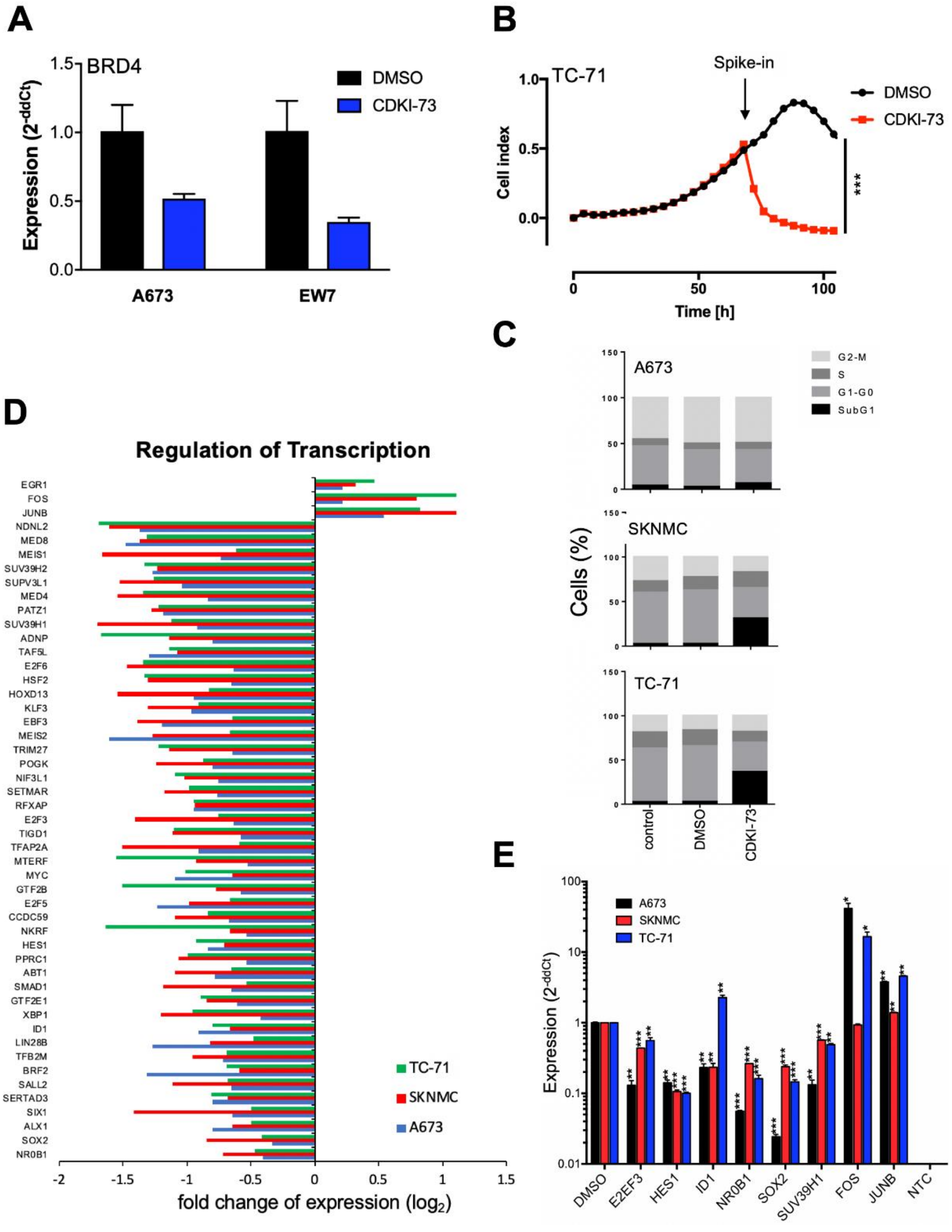

Supplementary Figure S2. Influence of CDK9 inhibition on transcriptional regulation and cell cycle progression. (**A**) Analysis of expression of BRD4 24 hrs after treatment with 2 μM CDKI-73 or DMSO in A673 and EW7 cell lines measured by qRT-PCR. Data are mean ± SEM. (**B**) TC-71 cells were treated with 2 μM CDKI-73 or DMSO as negative control. Cellular impedance was measured every 4 hrs (relative cell index). Data are mean ± SEM (hexaplicates/group); 2-way ANOVA. *** *p*-value <0.0005. (**C**) Cell cycle analysis of A673, SKNMC, or TC-71 cells 24 h post-treatment with 2 μM CDKI-73 or DMSO, respectively. Cells were stained with propidium iodide, and cell cycle effects were analyzed

by flow cytometry. (**D**) Expression array analysis of genes involved in the regulation of transcription differentially regulated after inhibition of CDK9 activity in EwS cell lines. (**E**) Verification of microarray data by qRT-PCR of selected, transcriptionally relevant genes. Data are mean ± SEM; *t*-test *** p-value < 0.0005, ** *p*-value < 0.005, * *p*-value < 0.05. The EwS specific transcription program was significantly de-regulated after CDKI-73 treatment in different EwS cell lines.

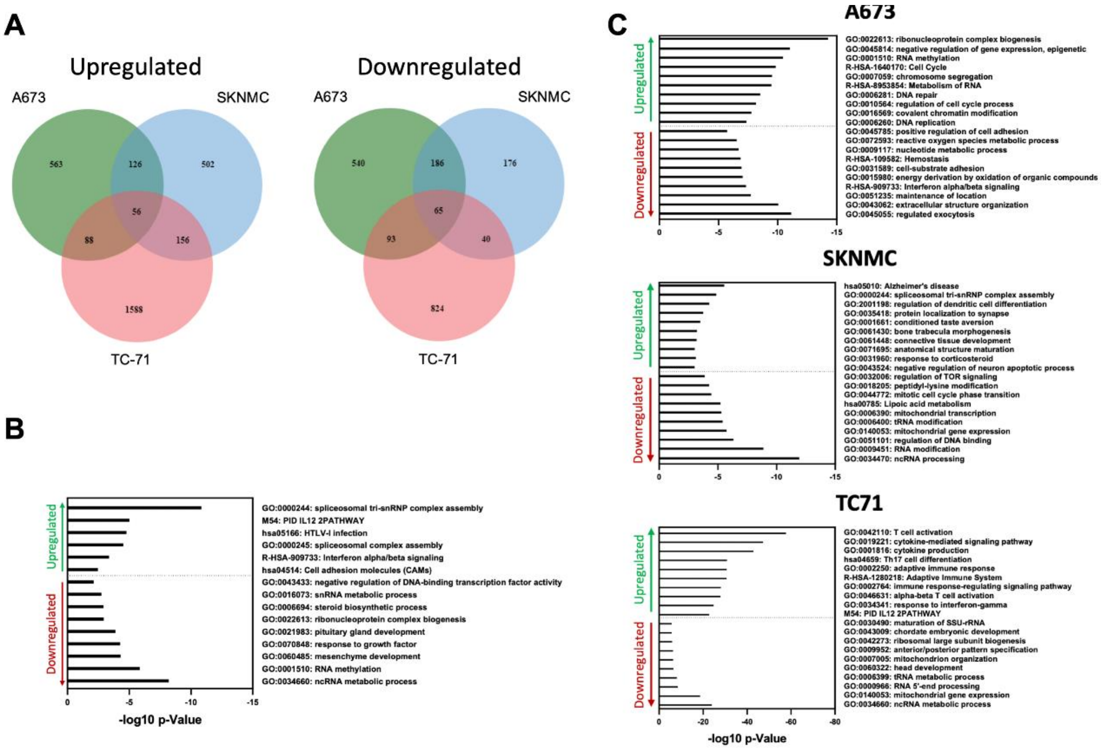

Supplementary Figure S3. GO Analysis of differentially regulated genes. The array data from Affymetrix humGene ST arrays performed with A673, SKNMC, and TC71 cell lines treated with CDKI-73 was RMA normalized (see Materials and Methods; GSE119546). A fold change cut-off of >1.5 for up-regulation and <0.5 for down-regulation was applied to the data. (**A**) The overlapping up- or down-regulated genes were identified using the jvenn platform [52]. The Venn diagram of up- and down-regulated genes are shown. (**B**) GO biological processes shared and deregulated in the three EwS lines as analyzed via the Metascape platform [53]. (**C**) Most deregulated biological processes in each individual EwS cell line.

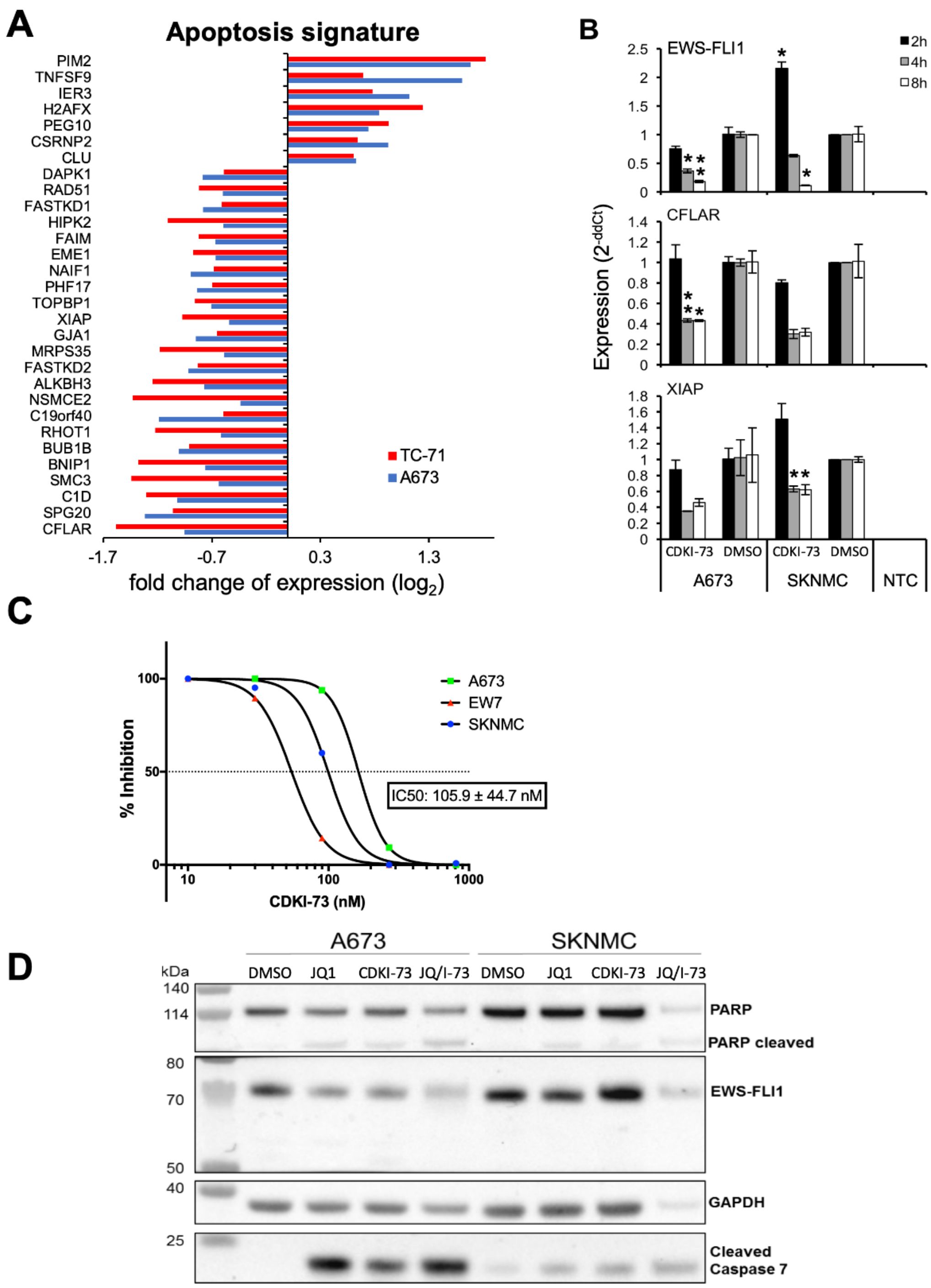

Supplementary Figure S4. Influence of CDK9 inhibition on the regulation of apoptosis. (**A**) Expression array analysis of genes involved in apoptosis signature differentially regulated after inhibition of JQ1 activity in EwS cell lines [18]. (**B**) Inhibition of CDK9 activity by 2 µM CDKI-73 down-regulated EWS-FLI1, CFLAR, and XIAP, genes important for survival of EwS cells, as analyzed by qRT-PCR analysis. Representative experiment (n = 3) shown; * p-value <0.05; ** p-value <0.005. (**C**) Dose-response curves at different concentrations of CDKI-73 (IC50 values, calculated for time point when control wells reached 90% confluence) in A673, SKNMC, or EW7 cells, respectively. (**D**) Western blot analysis of apoptosis susceptibility after JQ1 and/or CDKI-73 (I-73) treatment, respectively. Protein levels measured by antibodies against EWS-FLI1, PARP, CASP7, and GAPDH as loading control. A673 or SKNMC cells were treated for 48 h with inhibitors shown.

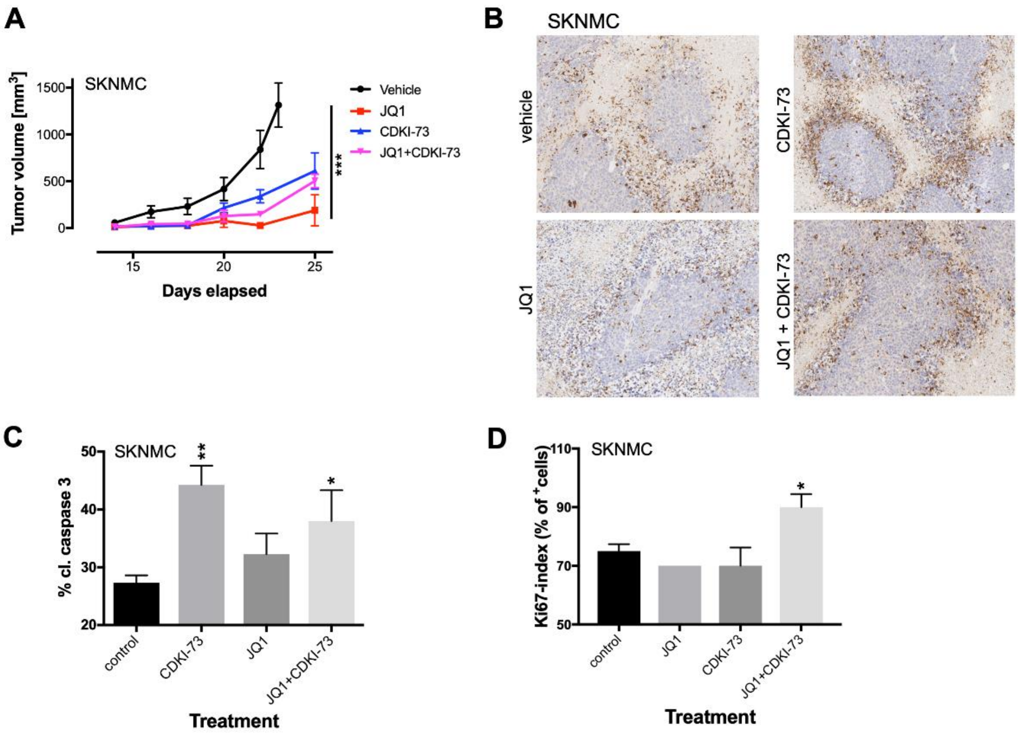

Supplementary Figure S5. Combined targeting of CDK9 and BRD4 of SKNMC tumors results in the inhibition of proliferation and tumor growth. (**A**) In vivo anti-tumor efficacy of JQ1 and CDKI-73 as a single agent or in combination against SKNMC-bearing Rag $2^{-/-}\gamma c^{-/-}$ mice xenografts. SKNMC cells were injected s.c. into mice. Once the tumor was palpable, mice were randomly allocated into four groups (6 mice / group) and treated with vehicle (DMSO), JQ1 (50 mg/kg, i.p., daily), CDKI-73 (50 mg/kg, p.o. every other day) or with CDKI-73 in combination with JQ1. For the combination study, JQ1 was given i.p. daily, followed by CDKI-73 every other day. Two-way ANOVA; *** *p*-value <0.0005. (**B**) Immunohistochemistry of tumors analyzed for cleaved caspase 3 expression. Results of representative SKNMC derived tumors are shown (4× original magnification). (**C**) The level of caspase 3 in SKNMC tumors. The percentage of cleaved caspase 3 positive cells in five fields per tumor is given; *t*-test, * *p*-value <0.05; ** *p*-value <0.005. (**D**) The percentage of Ki67 positive cells in five fields per tumor is given; *t*-test, * *p*-value <0.05.

Figure 1B and Figure S1B

Densitometry

	A673			SKNMC			TC-71		
	Input	IgG	CoIP	Input	IgG	CoIP	Input	IgG	CoIP
BRD4	1.00	0.05	1.88	1.00	-0.33	4.10	1.00	0.08	6.07
FLI1	1.00	-0.14	0.48	1.00	0.06	0.55	1.00	0.22	0.66
pCDK9	1.00	-0.03	1.23	1.00	0.37	1.49	1.00	-0.26	2.90
Tubulin	1.00	0.00	0.01	1.00	0.00	0.04	1.00	0.00	0.05
GAPDH	1.00	0.00	0.00	1.00	0.00	0.01	1.00	0.00	-0.04

Blot

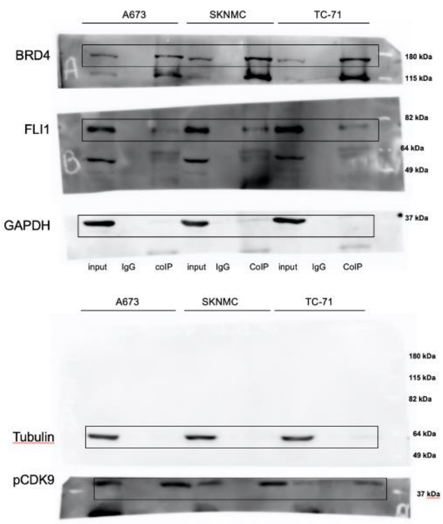

Figure S3D

Densitometry

	A673				SKNMC			
	DMSO	JQ1	CDKI-73	JQ1/I73	DMSO	JQ1	CDKI-73	JQ1/I73
PARP	1.00	0.69	0.92	0.55	1.00	1.00	1.15	0.06
Cleaved PARP	1.00	5.01	3.98	7.49	1.00	5.21	3.81	4.23
EWS-FLI1	1.00	0.46	0.52	0.36	1.00	0.84	1.46	0.14
GAPDH	1.00	0.87	0.92	0.63	1.00	1.16	1.27	0.10
Cleaved Caspase7	1.00	539.82	313.63	533.80	1.00	2.57	3.36	3.85

Blot

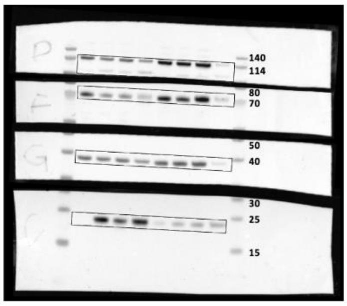

Figure 4C

Densitometry

	A673				SKNMC			
	DMSO	JQ1	I73	JQ1/I73	DMSO	JQ1	I73	JQ1/I73
PARP	1.00	0.85	0.86	0.91	1.00	1.14	1.08	0.13
Cleaved PARP	1.00	4.41	1.22	3.18	1.00	1.21	1.62	3.40
EWS-FLI1	1.00	0.77	0.40	0.68	1.00	0.81	1.28	0.16
GAPDH	1.00	0.83	0.75	0.57	1.00	1.06	0.88	0.09
Cleaved Caspase7	1.00	41.79	1.89	17.55	1.00	2.16	4.52	267.65

Blot

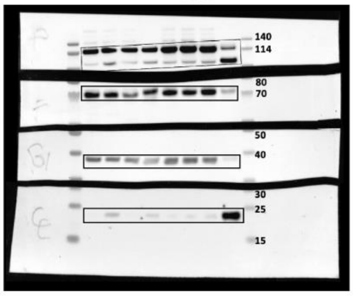

Supplementary Figure S6. Whole Western blots with molecular weight markers and densitometry of individual Western blot figures. Densitometry was either normalized to input (Figure 1B) or DMSO control (Supplementary Figure S3D and Figure 4C), respectively.

Figure S1B

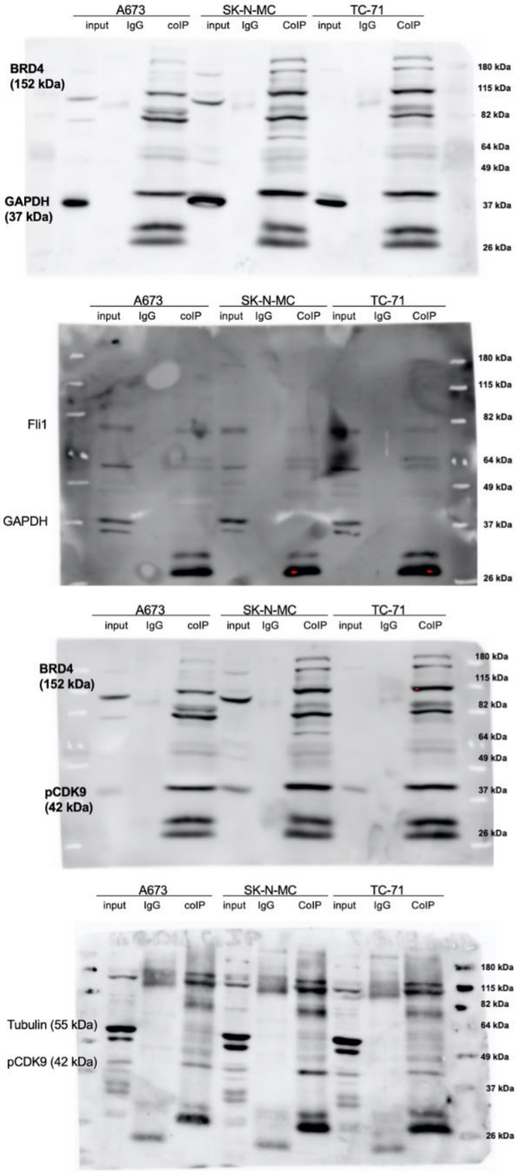

	A673			SKNMC			TC-71		
	Input	IgG	CoIP	Input	IgG	CoIP	Input	IgG	CoIP
BRD4	0.702	0	1.035	0.996	0	3.304	0.634	0	20.097
FLI1	0.946	0	0.1768	0.952	0	0.278	0.992	0	0.362
pCDK9	1	0	2.42	0.995	0	3.853	0.945	0	1.905
Tubulin	0.99	0	0.019	0.998	0	0.059	0.997	0	0.013
GABDH	0.999	0	0	1	0	0.021	1	0	0.02

Supplementary Figure S7. Whole Western blots with molecular weight markers for densitometry in Supplementary Figure S1B. Densitometry was normalized to input, and subsequently, IgG values were subtracted due to the absence of banding in IgG lanes.
